# TMC2 Modifies Permeation Properties of the Mechanoelectrical Transducer Channel in Early Postnatal Mouse Cochlear Outer Hair Cells

**DOI:** 10.3389/fnmol.2017.00326

**Published:** 2017-10-18

**Authors:** Laura F. Corns, Jing-Yi Jeng, Guy P. Richardson, Corné J. Kros, Walter Marcotti

**Affiliations:** ^1^Department of Biomedical Science, University of Sheffield, Sheffield, United Kingdom; ^2^Sussex Neuroscience, School of Life Sciences, University of Sussex, Brighton, United Kingdom

**Keywords:** sensory transduction, auditory, development, mechanoelectrical transduction, hair cells, TMC channels, dihydrostreptomycin

## Abstract

The ability of cochlear hair cells to convert sound into receptor potentials relies on the mechanoelectrical transducer (MET) channels present in their stereociliary bundles. There is strong evidence implying that transmembrane channel-like protein (TMC) 1 contributes to the pore-forming subunit of the mature MET channel, yet its expression is delayed (~>P5 in apical outer hair cells, OHCs) compared to the onset of mechanotransduction (~P1). Instead, the temporal expression of TMC2 coincides with this onset, indicating that it could be part of the immature MET channel. We investigated MET channel properties from OHCs of homo- and heterozygous *Tmc2* knockout mice. In the presence of TMC2, the MET channel blocker dihydrostreptomycin (DHS) had a lower affinity for the channel, when the aminoglycoside was applied extracellularly or intracellularly, with the latter effect being more pronounced. In *Tmc2* knockout mice OHCs were protected from aminoglycoside ototoxicity during the first postnatal week, most likely due to their small MET current and the lower saturation level for aminoglycoside entry into the individual MET channels. DHS entry through the MET channels of *Tmc2* knockout OHCs was lower during the first than in the second postnatal week, suggestive of a developmental change in the channel pore properties independent of TMC2. However, the ability of TMC2 to modify the MET channel properties strongly suggests it contributes to the pore-forming subunit of the neonatal channel. Nevertheless, we found that TMC2, different from TMC1, is not necessary for OHC development. While TMC2 is required for mechanotransduction in mature vestibular hair cells, its expression in the immature cochlea may be an evolutionary remnant.

## Introduction

The conversion of acoustic mechanical stimuli into electrical signals, known as mechanoelectrical transduction (MET), is essential for our sense of hearing and is performed by the stereociliary bundle, a staircase-like structure of actin–based protrusions found at the apex of cochlear hair cells. Within each hair bundle, stereocilia from different rows are connected by tip links in the direction of optimal mechanosensitivity of the hair bundle (Pickles et al., 1984; Assad et al., 1991). As the stereocilia are deflected towards the tallest row, the increased tension in the tip link increases the opening probability of the MET channel, which is found at the lower tip link insertion site (Beurg et al., 2009). The MET current, which is mainly carried by K^+^ but also by Ca^2+^ ions, generates depolarizing receptor potentials in hair cells, leading to the release of the neurotransmitter glutamate from ribbon synapses (Marcotti, 2012; Fettiplace and Kim, 2014).

The full molecular identity of the MET channel is still unknown; however, it is possible that the MET channel is not one molecule but a complex that can vary in composition with age and along the tonotopic axis of the cochlea (Kawashima et al., 2011; Zhao et al., 2014). Both TMHS/LHFPL5 and TMIE have been identified as components of the MET channel complex (Xiong et al., 2012; Zhao et al., 2014), but appear to be auxiliary subunits. Recent evidence has also shown that Piezo 1 and Piezo 2, which constitute the pore forming subunit of other mechanosensitive channels (Coste et al., 2010, 2012; Woo et al., 2014), do not contribute to the MET channel complex located at the tip of the outer hair cell (OHC) stereociliary bundles (Corns and Marcotti, 2016; Wu et al., 2017). Increasing evidence indicates that TMC1 could be the elusive pore-forming subunit of the MET channel (Kawashima et al., 2011; Pan et al., 2013; Kurima et al., 2015; Corns et al., 2016). The most compelling evidence for TMC1 being part of the pore-forming subunit, is that the single dominant missense mutation of a neutral methionine to a positively-charged lysine at position 412 of *Tmc1* (*Beethoven* mice: Kurima et al., 2002; Vreugde et al., 2002) affects the Ca^2+^ permeability and conductance of the MET channel (*Beethoven* mice: inner hair cells (IHCs): Pan et al., 2013; OHCs: Beurg et al., 2015; Corns et al., 2016), and the affinity for the permeant MET channel blocker dihydrostreptomycin (DHS), an aminoglycoside antibiotic, for its binding site within the permeation pore of the channel (Corns et al., 2016). Changes in Ca^2+^ permeability and DHS affinity of the anomalous MET channel have also been investigated in hair cells lacking both TMC1 and TMC2 (knockout mice: Kim et al., 2013; Beurg et al., 2014).

The expression level of *Tmc1* is almost undetectable at the onset of mechanotransduction (Kawashima et al., 2011) at around P1 in mouse apical OHCs (Lelli et al., 2009; Chen et al., 2014; Marcotti et al., 2014), and increases by about 20-fold by P5 (Kawashima et al., 2011). The expression profile of *Tmc1* suggests that additional molecules could form the MET channel pore at early postnatal stages. Another member of the TMC family, TMC2, has an mRNA expression pattern that correlates with the onset of mechanotransduction (Kawashima et al., 2011) and is localized to the tips of the middle and lower stereocilia (Kurima et al., 2015) where the MET channel is thought to reside. Although it has been shown that mechanotransduction can occur in the sole presence of TMC2 in neonatal hair cells (Kawashima et al., 2011), there is currently little evidence for its direct influence on the core MET channel properties. Here, we demonstrate that TMC2 confers a higher Ca^2+^ permeability to the MET channels and reduces the affinity of the MET channel for DHS, an effect that was more pronounced from the intracellular side. We also found that early postnatal OHCs from *Tmc2*^−/−^ mice were protected from aminoglycoside ototoxicity compared to control littermates. This protection from ototoxic damage is likely to be due to, in addition to the smaller MET current in *Tmc2*^−/−^ mice, the lower saturation level for aminoglycoside entry into the MET channel. Different from TMC1 (Marcotti et al., 2006), TMC2 was not required for OHC development. Our findings support a role for TMC2 as a pore forming subunit in the neonatal MET channel, prior to the expression of TMC1.

## Materials and Methods

### Ethical Approval

All experiments were performed in accordance with Home Office regulations under the Animals (Scientific Procedures) Act 1986 and following approval by the University of Sheffield Ethical Review Committee. *Tmc2* knockout mice were obtained from The Jackson Laboratory (B6.129-Tmc2^tm1.1Ajg^), maintained on a C57BL/6J background, and genotyped as previously described (Pan et al., 2013).

### Acute Tissue Preparation

OHCs were studied in acutely dissected organs of Corti from mice of postnatal day (P) 3 to P14. This wide age-range was used to investigate the role of TMC2, which is mainly expressed during the first postnatal week, in determining the biophysical and developmental properties of the MET current, and how these compare when TMC1 is present (mainly expressed from the second postnatal week) (Kawashima et al., 2011). We have also investigated whether the absence of TMC2 affected the normal progression of OHC development by measuring their basolateral membrane properties at immature (P6) and mature (P13–P14) stages.

In the mouse, experiments were performed on OHCs positioned in the apical coil of the cochlea, corresponding to a frequency range in adult animals of about 6–11 kHz (Müller et al., 2005). This cochlear region corresponds to about the position Ac-2 in Figure 2. We focused our experiments on apical OHCs because this cochlear region is easier to dissect in the mouse. In a few experiments, P7–P8 OHCs were also recorded from the apical region of the Mongolian gerbil cochlea (Figure 6), which is tuned to a characteristic frequency of ~0.35 kHz (Müller, 1996).

**Figure 1:**
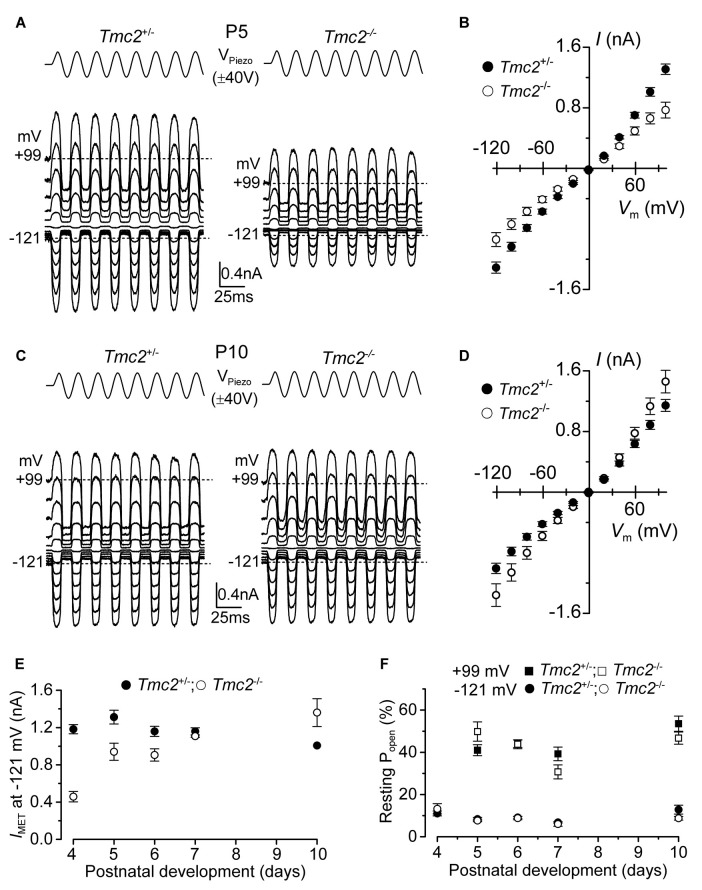
The absence of TMC2 does not impair the maturation of the mechanoelectrical transducer (MET) current.** (A)** Saturating MET currents in P5 apical outer hair cells (OHCs) from control *Tmc2*^+/−^ (left) and knockout *Tmc2*^−/−^ (right) mice in response to a 50 Hz sinusoidal force stimulus to the hair bundles at membrane potentials from −121 mV to +99 mV in 20 mV nominal increments. Dashed lines indicate the holding current at −121 mV and +99 mV. V_Piezo_ indicates the driver voltage to the fluid jet, with positive deflections moving the hair bundles in the excitatory direction. **(B)** Peak-to-peak MET current-voltage curves obtained from 14 *Tmc2*^+/−^ and 10 *Tmc2*^−/−^ OHCs at P5. **(C)** Saturating MET currents obtained as described in **(A)** but from P10 apical OHCs of *Tmc2*^+/−^ (left) and *Tmc2*^−/−^ (right) mice. **(D)** Peak-to-peak MET current-voltage curves obtained from 6 *Tmc2*^+/−^ and 6 *Tmc2*^−/−^ OHCs at P10. **(E)** Saturating MET currents at the membrane potential of −121 mV recorded from P4 to P10 apical OHCs from both genotypes. The size of the MET current was found to be significantly different between the two genotypes only at P4 (*P* < 0.0001) and P5 (*P* < 0.001) – 2-way ANOVA. Number of cells from left to right are: *Tmc2*^+/−^ 15 (5 mice), 14 (5 mice), 19 (9 mice), 10 (4 mice), 6 (3 mice); *Tmc2*^−/−^ 5 (1 mouse), 10 (3 mice), 4 (3 mice), 3 (2 mice), 6 (2 mice).** (F)** Resting open probability (*P*_open_) of the MET channel at −121 mV and +99 mV from OHCs of *Tmc2*^+/−^ and *Tmc2*^−/−^ mice. Number of OHCs tested at both membrane potentials are: *Tmc2*^+/−^ 8, 14, 19, 10, 4; *Tmc2*^−/−^ 5, 10, 4, 3, 3. Number of mice as in panel **(E)**. Note that at P4 only values at −121 mV were obtained.

**Figure 2:**
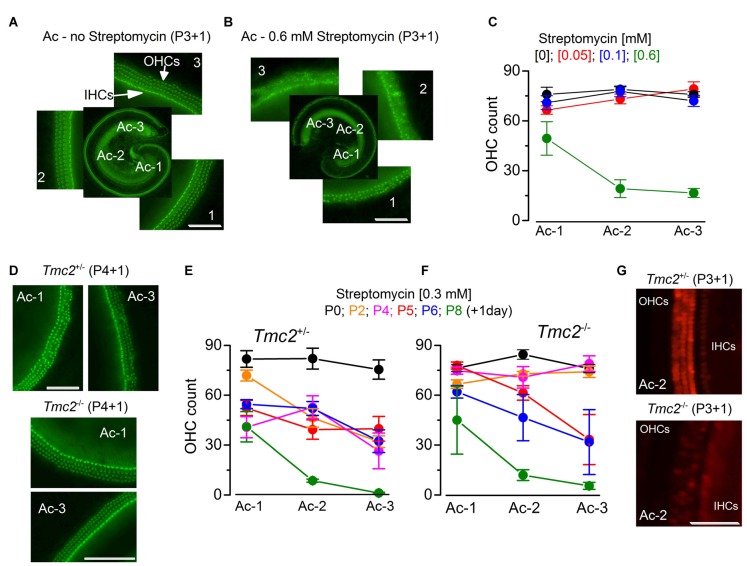
The absence of TMC2 protects OHCs from aminoglycoside ototoxicity **(A,B)**. Fluorescence images from the apical coil of the cochlea of P3 + 1 day *in vitro* C57B mice taken after incubation of the tissue with Alexa Fluor 488 phalloidin without **(A)** or following 24 h exposure to 0.6 mM streptomycin **(B)**. Note that in the expanded panels, the hair bundles of both inner hair cells (IHCs; one row) and OHCs (3 rows) are clearly visible in **(A)**, but almost completely absent after streptomycin treatment **(B)**. The numbers indicate the position along the apical coil from apical (Ac-1) to middle (Ac-2) and base (Ac-3). Scale bars, 100 μm. **(C)** Number of OHCs present in a 200 μm length of the sensory epithelium from the three different cochlear positions highlighted in **(A,B)**, in the absence (: 14 cochleae, 8 mice) or presence of the different [mM] concentrations of streptomycin ([0.05]: 8 cochleae, 4 mice; [0.1]: 9 cochleae, 5 mice; [0.6]: 5 cochleae, 3 mice). Note that the presence of bundles was used as an indication for the presence of hair cells. **(D)** Fluorescence images (as in **(A,B)** obtained from P4 + 1 day *in vitro* cochleae of *Tmc2*^+/−^ (top) and *Tmc2*^−/−^ (bottom) mice in the presence of 0.3 mM streptomycin. Scale bars, 200 μm. **(E,F)** Number of OHCs in *Tmc2*^+/−^ **(E)** and *Tmc2*^−/−^ **(F)** obtained as described in **(C)** but after incubating cochleae with 0.3 mM streptomycin at different postnatal ages (P0, P2, P4, P5, P6 and P8; all + 1 day *in vitro*). Number of cochleae and mice tested were: 5 and 3 (P0); 6 and 3 (P2); 8 and 5 (P4); 11 and 7 (P5); 12 and 6 (P6); 6 and 3 (P8) in **(E)**; 8 and 4 (P0); 8 and 5 (P2); 9 and 5 (P4); 6 and 5 (P5); 5 and 3 (P6); 4 and 2 (P8) in **(F)**. Differences between the two genotypes were tested for statistical significance with 2-way ANOVA: P0 (n.s), P2 (*P* < 0.0001), P4 (*P* < 0.0001), P5 (*P* = 0.027), P6 (n.s), P8 (n.s). **(G)** Fluorescence images of hair cells labeled with Texas Red neomycin from P3 + 1 day *in vitro* cochleae of *Tmc2*^+/−^ (top) and *Tmc2*^−/−^ (bottom) mice; cochleae were incubated for 5 min in ~0.1 mM Texas Red neomycin. Scale bars, 200 μm.

**Figure 3:**
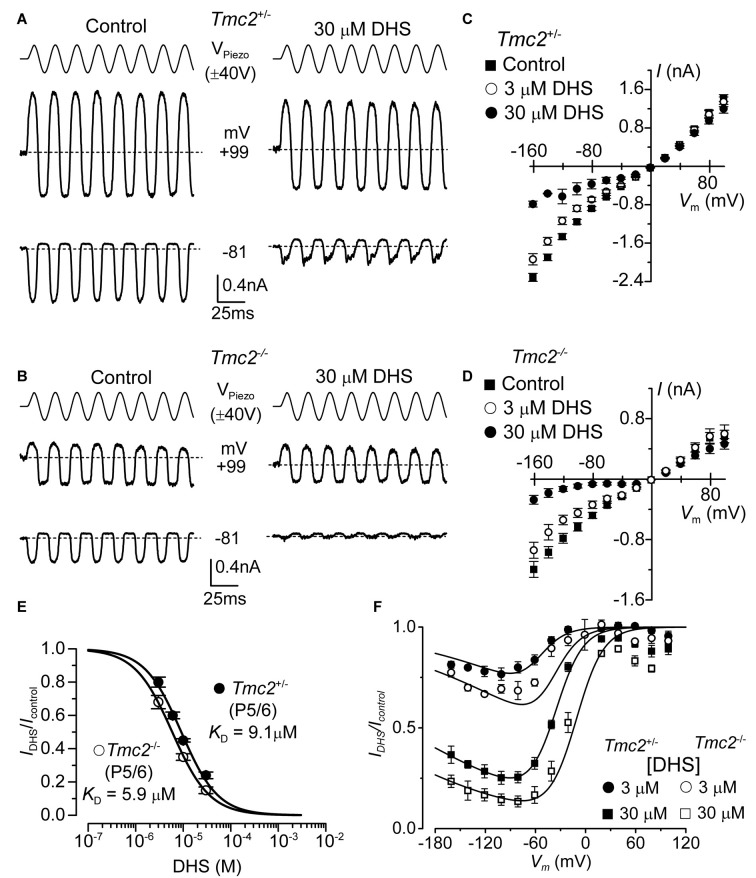
The affinity of extracellularly applied dihydrostreptomycin (DHS) for the MET channel is increased in *Tmc2*^−/−^ OHCs.** (A,B)** Saturating MET currents from apical OHCs of *Tmc2*^+/−^ **(A)** and *Tmc2*^−/−^ **(B)** mice in response to sinusoidal stimuli to the hair bundles at membrane potentials of –81 mV and +99 mV in control (left) and 30 μM DHS (right) extracellular solution. **(C,D)** Average peak-to-peak MET current-voltage curves recorded from P5–P6 apical OHCs from *Tmc2*^+/−^ **(C)** and *Tmc2*^−/−^ **(D)** mice in control conditions, 3 μM and 30 μM extracellular DHS. Number of OHCs recorded are: *Tmc2*^+/−^ control (*n* = 13, 5 mice), 3 μM DHS (*n* = 8, 4 mice), 30 μM DHS (*n* = 3, 1 mouse); *Tmc2*^−/−^ control conditions (*n* = 6, 4 mice), 3 μM DHS (*n* = 3, 2 mice), 30 μM DHS (*n* = 5, 4 mice). **(E)** Dose-response curves for the block of the MET current by extracellular DHS at –81 mV in OHCs from *Tmc2*^+/−^ (closed symbol) and *Tmc2*^−/−^ (open symbol). Data were fitted using the Hill equation: *Tmc2*^+/−^ OHCs (P5–P6) half-blocking concentration *K*_D_ 9.1 ± 0.4 μM and Hill coefficient *n*_H_ 1.12 ± 0.08 (number of OHCs: 8, 4 mice, 8, 3 mice, 8, 3 mice, 5, 3 mice); *Tmc2*^−/−^ OHCs (P5–P6) *K*_D_ 5.9 ± 0.2 μM and *n*_H_ 1.10 ± 0.04 (number of OHCs: 8, 4 mice, 8, 4 mice, 10, 6 mice). **(F)** Voltage-dependent block of the MET current by DHS obtained by plotting its size in the presence of different concentrations of the aminoglycoside as a fraction of the current in the control solution (*I*_DHS_/*I*_C_). Number of cells is as in panel **(C)** (*Tmc2*^+/−^) and **(D)** (*Tmc2*^−/−^). Continuous lines are fits according to the two-barrier one-binding-site model (see “Materials and Methods” section). The fitted parameters are: Δ*δ* = *δ*_2_ − *δ*_1_: 0.91 and *δ*_b_: 0.79 for all conditions tested; Δ*E* was 4.555 *kT* in *Tmc2*^+/−^ and 3.452 *kT* in *Tmc2*^−/−^; *E*_b_ was –8.035* kT* in *Tmc2*^+/−^ and –9.637* kT* in *Tmc2*^−/−^. The fractional block was significantly different at *P* < 0.01 (2-way ANOVA) for both genotypes at each concentration.

**Figure 4:**
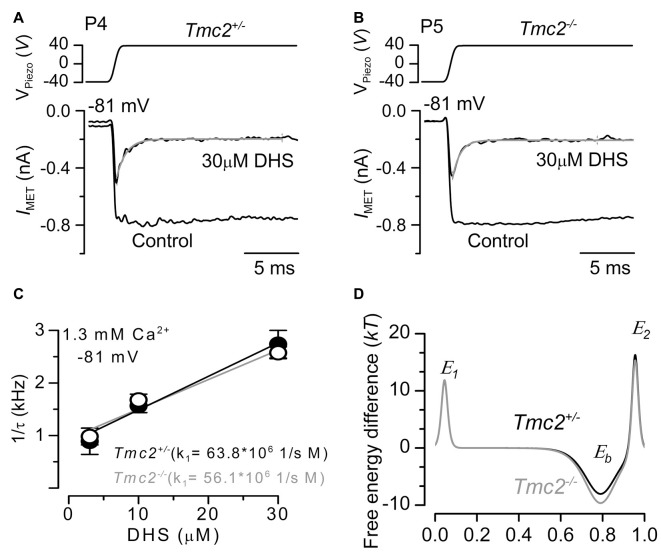
The rate of entry of DHS into the hair cells is not affected in *Tmc2*^−/−^ mice. **(A,B)** Saturating MET currents recorded from the apical OHCs of P4 control *Tmc2*^+/−^ **(A)** and P5 knockout *Tmc2*^−/−^ **(B)** mice before and during the application of 30 μM extracellular DHS. Bundles were deflected in response to step stimuli (top panel) and at membrane potentials of –81 mV. MET channels were first closed by inhibitory bundle displacement and then fully opened by an excitatory deflection. The decay of the MET current in the presence of 30 μM DHS was fitted using a single exponential (*Tmc2*^+/−^: *τ* = 0.45 ms; *Tmc2*^−/−^: *τ* = 0.36 ms). **(C)** The inverse of the time constant of binding (1/*τ*), which was obtained by the experiments shown in panels **(A,B)** (see “Results” section), was plotted against three different extracellular DHS concentrations in each genotype. Solid lines indicate the fits and the slope *k*_1_ is indicated for *Tmc2*^+/−^ and *Tmc2*^−/−^ OHCs. Number of OHCs (P5–P6) from left to right: 3 (2 mice), 8 (3 mice), 6 (3 mice) for *Tmc2*^+/−^; 8 (3 mice), 8 (4 mice), 7 (3 mice) for *Tmc2*^−/−^. **(D)** Energy profile of two barrier–one binding site model for the MET-channel pore of* Tmc2*^+/−^ (black) and *Tmc2*^−/−^ (gray) OHCs. In the absence of a voltage across the membrane (*V*_m_ = 0), the two barriers have estimated free energies *E*_1_ (11.8 *kT* for controls and 11.9 *kT* for *Tmc2* knockouts) and *E*_2_ (16.3 *kT* for controls and 15.3 *kT* for *Tmc2* knockouts). The barriers are located at relative electrical distances *δ*_1_ of 0.045 and *δ*_2_ of 0.955, as measured across the membrane from the extracellular side. The two barriers sandwich the binding site for DHS at a relative electrical distance *δ*_b_ of 0.79 with a minimum in free energy, *E*_b_ of −8.04 *kT* for controls and −9.64 *kT* for *Tmc2* knockouts.

**Figure 5:**
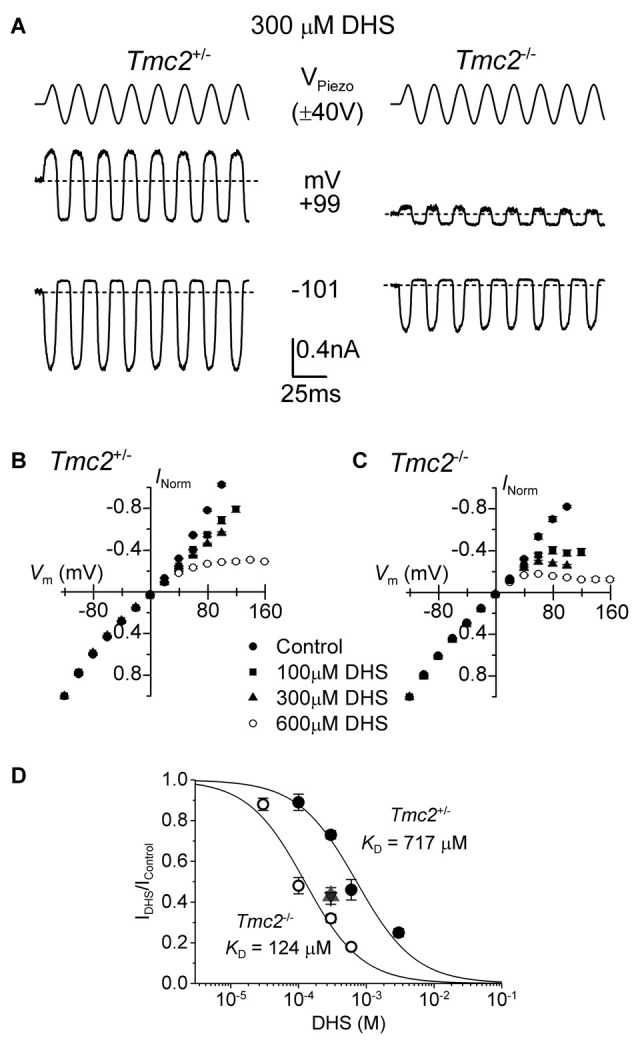
The block of the MET channel by intracellular DHS is largely increased in OHCs from *Tmc2* knockout mice. **(A)** Saturating MET currents in response to sinusoidal stimuli at –101 mV and +99 mV from apical OHCs of *Tmc2*^+/−^ (left) and *Tmc2*^−/−^ (right) mice in the presence of 300 μM DHS in the intracellular solution. Note that intracellular DHS blocks the MET current at depolarized potentials in OHCs from both genotypes. **(B,C)** Average normalized MET current-voltage response curves from P4 apical OHCs of *Tmc2*^+/−^ and *Tmc2*^−/−^ mice, respectively, in control condition and in the presence of different intracellular concentrations of DHS. Numbers of OHCs recorded under the different conditions were: *Tmc2*^+/−^ control conditions (*n* = 8), 100 μM DHS (*n* = 4), 300 μM DHS (*n* = 15) and 600 μM DHS (*n* = 3); *Tmc2*^−/−^ control conditions (*n* = 4), 100 μM DHS (*n* = 5), 300 μM DHS (*n* = 5) and 600 μM DHS (*n* = 5). **(D)** Dose-response curves for the block of the MET current by intracellular DHS at +99 mV in P4 OHCs from *Tmc2*^+/−^ (closed symbols) and *Tmc2*^−/−^ (open symbols). Continuous lines are fits through the data using the Hill equation. *Tmc2*^−/−^ OHCs (P4-P5) *K*_D_ 124 ± 12 μM (number of OHCs from left to right: 4 (1 mouse), 15 (5 mice), 3 (2 mice), 6 (4 mice)). *Tmc2*^+/−^ OHCs (P4–P5) *K*_D_ 717 ± 60 μM (number of OHCs: 5 (2 mice), 5 (3 mice), 5 (1 mice), 5 (2 mice)). η_H_ was 1.0 in both genotypes. The degree of block in P8 OHCs, which was only done at one concentration (300 μM DHS) as a comparison with P4–P5, is shown by the triangle symbol (note the overlapping response of *Tmc2*^+/−^ (*n* = 7, 2 mice) and *Tmc2*^−/−^ (*n* = 7, 2 mice) OHCs).

**Figure 6:**
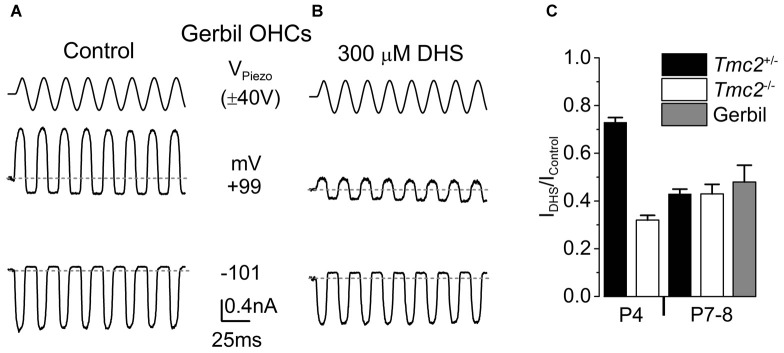
The degree of block by intracellular DHS increases with age in gerbil and mouse apical OHCs. **(A,B)** Saturating MET currents in response to sinusoidal stimuli at –101 mV and +99 mV from apical OHCs of a P8 **(A)** or P7 **(B)** gerbil, using Cs-glutamate based intracellular **(A)** or Cs-glutamate based intracellular plus 300 μM DHS **(B)**. **(C)** Normalized responses showing the degree of block of the MET current at +99 mV by 300 μM intracellular DHS in apical OHCs from *Tmc2*^+/−^ and *Tmc2*^−/−^ mice at P4 and P8, and gerbils at P7–P8. The degree of block in P4 *Tmc2*^+/−^ OHCs was found to be significantly reduced (*P* < 0.001) compared to all other conditions. No other significant differences were found. Number of OHCs: 15 (5 mice), 5 (1 mouse), 7 (2 mice), 7 (2 mice), 4 (3 gerbils).

Mice and gerbils of either sex were killed by cervical dislocation, the cochlea removed and the organ of Corti dissected in extracellular solution composed of (in mM): 135 NaCl, 5.8 KCl, 1.3 CaCl_2_, 0.9 MgCl_2_, 0.7 NaH_2_PO_4_, 5.6 D-glucose, 10 HEPES-NaOH, 2 Na-pyruvate. Amino acids and vitamins (Eagle’s MEM) were added from concentrate (pH 7.5, 308 mOsmol/kg). Once dissected, the apical coils of the organs of Corti were transferred to a microscope chamber filled with extracellular solution and viewed using a long working-distance 63× water-immersion objective on a Leica DMLFS microscope.

### Whole Cell Patch Clamp

Soda glass capillaries were used to pull patch pipettes of resistances 2–4 MΩ and the shank of the electrode was coated in surf wax (Mr. Zoggs Sex Wax, CA, USA). Pipettes were filled with an intracellular solution of composition (in mM): 106 L-glutamic acid, 20 CsCl, 10 Na_2_phosphocreatine, 3 MgCl_2_, 1 EGTA-CsOH, 5 Na_2_ATP, 5 HEPES and 0.3 GTP (pH adjusted to 7.3 with CsOH, 294 mOsmol/kg) for most MET current recordings except those measuring Ca^2+^ permeability. For basolateral recordings, pipettes were filled with the following solution (in mM): 131 KCl, 3 MgCl_2_, 1 EGTA-KOH, 5 Na_2_ATP, 5 Hepes-KOH, 10 Na-phosphocreatine (pH 7.28, 298 mmol/kg). An Optopatch amplifier (Cairn Research Ltd., UK) was used to obtain MET current recordings at room temperature (20–25°C). pClamp software (Molecular Devices, USA: RRID:SCR_011323) and a Digidata 1440A were used to acquire data. Data were filtered at either 2.5 or 5 kHz (8-pole Bessel) and sampled at 10 or 50 kHz. Origin software (OriginLab, USA: RRID:SCR_002815) was used to perform offline data analysis. Membrane potentials were corrected for a measured liquid junction potential of −11 mV and −4 mV for Cs-glutamate and KCl intracellular solutions, respectively.

### Hair Bundle Stimulation

A 25 mm diameter piezoelectric disc was used to direct a fluid jet via a pipette onto the stereociliary bundle, displacement of which elicited the MET currents (Kros et al., 1992; Corns et al., 2014; Corns and Marcotti, 2016). The tip of the fluid jet pipette had a diameter of 8–10 μm and was positioned at about 8 μm from the bundles. Bundle displacement was calculated from previously calibrated recordings (10.1 nm/V: Corns et al., 2014). Mechanical stimuli were applied as steps or 50 Hz sinusoids (filtered at 1 kHz, 8-pole Bessel).

### Calcium Selectivity

The Ca^2+^ selectivity of the MET channel was determined under conditions where Ca^2+^ was the only permeant cation in the extracellular solution, and Cs^+^ was present in the intracellular solution. The reversal potential of the MET current, which was interpolated by fitting the data around the 0-current level with a linear regression line, was used to calculate the relative permeability, P_Ca_/P_Cs_ (see also Kim and Fettiplace, 2013). For these experiments, MET currents were recorded in response to sinusoidal 50 Hz force stimulation of hair bundles combined with a voltage ramp (288 mV/s) from −129 mV to +91 mV (see Corns et al., 2016). To measure the calcium reversal potential, a CsCl based intracellular solution of the following composition (mM): 135 CsCl, 3 MgATP, 10 Tris phosphocreatine, 1 EGTA-CsOH, 10 HEPES-CsOH (pH 7.2, 293 mOsmol/kg) and a high Ca^2+^ extracellular solution containing (mM): 100 CaCl_2_, 20 *N*-methylglucamine, 6 Tris, 10 D-glucose (pH 7.4), were applied as described above. A liquid junction potential of −9 mV was used to correct the reversal potentials. To calculate the relative permeability, P_Ca_/P_Cs_, the Goldman-Hodgkin-Katz equation was applied: P_Ca_/P_Cs_ = {a_1_[Cs^+^]/4a_2_[Ca^2+^]} × {exp(V_rev_F/RT)} × {1 + exp (V_rev_F/RT)}, where RT/F has its usual meaning with a value at room temperature of 25.7 mV, [Cs^+^] and [Ca^2+^] are the concentrations of Cs^+^ intracellularly (140 mM) and Ca^2+^ extracellularly (100 mM) and a_1_ (0.711) and a_2_ (0.519) are the published activity coefficients for Cs^+^ (Partanen, 2010) and Ca^2+^ (Rard and Clegg, 1997), respectively.

### Acute Dihydrostreptomycin Application

Stock solutions of 100 mM DHS (Sigma, UK; molecular weight = 730.7) were prepared in water and diluted to the final concentration in either extracellular or intracellular solution on the experimental day. During the recordings, all test solutions containing DHS were superfused via a pipette positioned orthogonally to the axis of mechanical sensitivity of the hair bundle and care was taken to ensure they were present in the fluid jet (for more details see: Corns et al., 2014; Corns and Marcotti, 2016).

### Two-Barrier One-Binding-Site Model of DHS Blockage of the MET Channel

The voltage-dependent block of the transducer current by extracellular DHS was quantitatively described by a two-barrier one-binding-site model previously described in more detail (Marcotti et al., 2005; van Netten and Kros, 2007), according to the reaction scheme:

$$C+D_{\text{0}}\begin{array}{c} k_{1}\\ \Leftrightarrow \\ k_{-1} \end{array}CD\begin{array}{c} k_{2}\\ \Leftrightarrow \\ k_{-2} \end{array}C+D_{\text{i}}$$

where *C* represents the unblocked MET channels, *CD* the blocked channels, *D*_0_ and *D*_i_ the extra- and intracellular blocker. The forward (*k*_1_, *k*_2_) and backward (*k*_−1_, *k*_−2_) rate constants are voltage dependent. In brief, the voltage dependence of the block is expressed by four parameters: *E*_b,_ the free energy of the drug binding site at zero membrane potential; *δ*_b_, the site’s fractional electrical distance across the membrane from the extracellular side; Δ*E* = *E*_2_ − *E*_1_, the difference between the intracellular and extracellular free energy levels of the two barriers at zero membrane potential; Δ*δ* = *δ*_2_ − *δ*_1_, the fractional electrical distance between the intra- and extracellular barriers. Fits using this model with a Hill coefficient, n_H_, of one and a valence for DHS of +2 (Marcotti et al., 2005) yield values for Δ*E*, *E*_b_, Δ*δ* and *δ*_b_, where the binding site is located at a relative electrical distance *δ*_b_ of 0.79 and Δ*δ* is 0.91 (Marcotti et al., 2005). The forward rate constant *k*_1_ equals the slope of τ^−1^ vs. [D]_0_, allowing calculation of the absolute values of the energy barriers *E*_1_ and *E*_2_. This in turn enabled us to calculate *k*_2_ and the entry rate of drug molecules into the OHCs.

### Cochlear Culture Preparation

Cochlear cultures from homozygous *Tmc2* knockout mice, their heterozygous littermate controls and wild-type C57BL/6J mice were prepared as described previously (Richardson and Russell, 1991). Briefly, cochleae were dissected from 3 day postnatal pups in HEPES buffered (10 mM, pH 7.2) Hanks’ balanced salt solution (HBHBSS), placed onto collagen-coated glass coverslips, fed one drop of complete medium (containing 93% DMEM/F12 (Sigma, UK), 7% fetal bovine serum (FBS: Biosera, UK) and 10 μg/ml ampicillin (Sigma)), sealed into Maximow slide assemblies and maintained at 37°C for 1 day.

### Streptomycin Treatment and Phalloidin Staining of the Mouse Cochlea

After a day at 37°C (see above, “Cochlear Culture Preparation” section), cochlear cultures were transferred into a sterile 35 mm-diameter petri dish (Corning) and incubated in a medium (100% DMEM/F12 and 10 μg/ml ampicillin) containing different concentrations of the aminoglycoside antibiotic streptomycin (Sigma) for 24 h at 37°C. A stock solution of streptomycin (100 mM; molecular weight 728.7) was prepared in HBHBSS. After 24 h, the coverslips with adherent cultures were washed three times with 3 ml of HBHBSS, fixed with 4% paraformaldehyde in phosphate buffered saline (PBS) for 1 h at 22°C, washed three times with PBS and stained with a solution containing Alexa Fluor 488 phalloidin (1:300: Life Technologies, RRID:AB_2315147), 0.7% FBS and 0.01% Triton -X100 for 2 h. The coverslips were then washed another three times in PBS, and the collagen with the attached cochleae was peeled off from the coverslips and mounted in Vectashield mounting medium (Vector Laboratoris, RRID:AB_2336789). Cochleae were imaged with an Olympus BXB61 with 10× or 20× dry objectives, and images were captured using the Volocity 3D Image Analysis Software (RRID:SCR_002668). The number of hair cells along the four different cochlear regions (see “Results” section) was measured over a 200 μm length region using Photoshop.

### Texas-Red Neomycin Accumulation in Hair Cells

Texas Red conjugated neomycin was prepared using a method similar to that described (Steyger et al., 2003) for producing a Texas Red conjugated derivative of gentamicin. In brief, 4.5 ml of neomycin sulfate (Sigma, 50 mg/ml 100 mM in K_2_CO_3_ buffer pH 8.5) was added to 0.5 ml of Texas Red succimidyl esters (mixed isomers, Invitrogen T6134, 2 mg/ml in dry dimethyl formamide) and the solution was mixed overnight on a rotator at 4°C. Aliquots were then snap frozen and used without further purification. Cultures were incubated in HBHBSS containing ~0.1 mM Texas Red neomycin for 5 min at 22°C, fixed in 4% PFA for 1 h, washed three times in PBS and labeled with phalloidin as described above. Images were captured using an Olympus BXB61 (see above). Texas Red neomycin was used for these experiments rather than Texas Red streptomycin as it produced more reproducible hair-cell loading during acute application. As a control for the stability of neomycin during conjugation, 4.5 ml of the neomycin solution was mixed with 0.5 ml dimethylformamide (DMF) overnight, and as a control for the specificity of the Texas Red neomycin conjugate, 0.5 ml of the Texas Red ester was mixed with 4.5 ml of the K_2_CO_3_ buffer. The neomycin retained toxicity after overnight incubation in the presence of 10% DMF, and labeling of hair cells was not observed with Texas Red alone. Hair-cell labeling was only observed with the Texas Red neomycin conjugate. In total, six control and four mutant cochlear cultures were examined from five mice.

### Statistical Analysis

Statistical analysis was performed with Prism (GraphPad Software, RRID:SCR_002798). Comparisons of means were made by Student’s two-tailed *t* test or deduced from the lower and upper confidence limits. For multiple comparisons ANOVA was used (one-way ANOVA followed by Tukey’s test; two-way ANOVA followed by Bonferroni’s test). *P* < 0.05 was selected as the criterion for statistical significance. All values are quoted as mean ± SEM. For most of the experiments the number of OHCs (or cochleae) and mice used is listed in the Figure legend. Note that in most cases both cochleae were used from each mouse.

## Results

The role of TMC2 in mechano-electrical transduction, and more generally on hair cell physiology, has received less attention than the closely related TMC1. Therefore, we have performed a set of experiments to provide more insights into the biophysical and developmental contribution of TMC2 to mechano-electrical transduction in cochlear outer hair cells (OHCs).

### Absence of TMC2 Does Not Prevent the Acquisition of Mature MET Channel Properties

Saturating MET currents from apical-coil OHCs of control (*Tmc2*^+/−^) and littermate mutant (*Tmc2*^−/−^) mice were elicited by displacing their hair bundles with sinewave stimuli from a piezoelectric fluid jet stimulator (Kros et al., 1992; Corns et al., 2014; Corns and Marcotti, 2016). At negative membrane potentials, bundle displacement in the excitatory direction (i.e., towards the taller stereocilia) elicited large inward MET currents from postnatal day 5 (P5) OHCs of both genotypes (Figures 1A,B). By this age, the expression of *Tmc2* mRNA in apical OHCs has slightly declined to ~75% of its maximum, which occurs at P4 (Kawashima et al., 2011). Membrane depolarization caused the MET current to decrease in size at first and then reverse near 0 mV to become outward at positive potentials (Figures 1A,B). As previously reported (Kim and Fettiplace, 2013), the maximum amplitude of the MET current was significantly reduced (*P* < 0.0001) in apical OHCs of *Tmc2*^−/−^ compared to* Tmc2*^+/−^ mice at P4 (by 61%) and P5 (by 28%; Figure 1E). Taking a value of 62 pS as the conductance of the MET channel for both genotypes (Kim et al., 2013), we infer the presence of some 156 MET channels in the *Tmc2*^+/−^ OHCs at P4, but only 60 in the *Tmc2*^−/−^ OHCs, suggesting a limiting supply of TMC1 protein at this stage. However, we also found that from P6 onward the MET current amplitude was comparable between the two genotypes (Figures 1C–E); this is because from around the end of the first postnatal week TMC1 is becoming the main TMC protein expressed in OHCs (Kawashima et al., 2011; Kim and Fettiplace, 2013) and the absence of TMC2 did not impair the normal development of the MET current. In contrast to recent observations in OHCs from mice with a point mutation in *Tmc1* (Corns et al., 2016), the resting open probability of the MET channel at both negative and positive membrane potentials was not affected by the absence of TMC2 (Figure 1F), indicating that the latter is not required for regulating the Ca^2+^ sensitivity of the MET channel’s adaptation sensor. We also found that during the second postnatal week, the Ca^2+^ permeability (see also “Materials and Methods” section for details) of the MET channel was similar in *Tmc2*^+/−^ (P_Ca_/P_Cs_: P6–P7 3.37 ± 0.17, *n* = 7; P9 3.10 ± 0.13, *n* = 4) and *Tmc2*^−/−^ (P6–P7 2.98 ± 0.12, *n* = 6; P9 3.27 ± 0.16, *n* = 5) OHCs. Despite this similarity at older ages, the Ca^2+^ permeability was significantly reduced in *Tmc2*^−/−^ P3–P4 OHCs (*Tmc2*^+/−^: 4.40 ± 0.24, *n* = 4; *Tmc2*^−/−^: 2.52 ± 0.13, *n* = 5, *P* < 0.0005), in agreement with previous findings (Kim and Fettiplace, 2013; Pan et al., 2013; Beurg et al., 2015).

### Effects of Streptomycin on OHC Survival

We further tested the MET channel properties in *Tmc2*^−/−^ OHCs by evaluating the permeation of larger molecules such as aminoglycoside antibiotics, which are known to behave as permeant blockers of the channel (Marcotti et al., 2005) leading to hair cell apoptosis (Forge and Schacht, 2000; Schacht et al., 2012). Initially, we tested the effect of streptomycin on cochlear hair cells from P3 C57BL/6J wild-type mice (Figures 2A–C), in order to determine the optimal concentration to be used on OHCs from *Tmc2*^−/−^ mice. Following 1 day in culture, we exposed the organs of Corti of C57BL/6J wild-type mice for 24 h to three different concentrations of streptomycin and found that while 0.1 mM had very little effect on hair-cell survival, 0.6 mM almost completely abolished all apical-coil OHCs (Figures 2A–C). Therefore we decided to use an intermediate concentration of streptomycin (0.3 mM) for the following experiments on *Tmc2*^−/−^ mice (Figures 2D–G). Streptomycin was tested on apical-coil OHCs at different postnatal ages (P0, P2, P4, P5, P6 and P8) in order to correlate the effect of streptomycin with the temporal expression of TMC2 and TMC1. We found that during a time when *Tmc2* is the major subunit expressed in the cochlea (P2–P4: Kawashima et al., 2011), OHCs from *Tmc2*^−/−^ mice were protected from the ototoxic effects of the aminoglycoside (Figures 2D,F) compared to those from *Tmc2*^+/−^ mice (Figures 2D,E). This protection was most likely due to, at least in part, the reduced number of MET channels, and hence reduced resting MET current in *Tmc2*^−/−^ OHCs. This interpretation was also supported by the reduced Texas Red-neomycin hair-cell labeling of *Tmc2*^−/−^ compared to that of *Tmc2*^+/−^ organs of Corti at this early age (Figure 2G). Although the reduced labeling in *Tmc2*^−/−^ mice was not quantified, the same result as that shown in Figure 2G was seen in a total of 12 cochleae from six *Tmc2*^+/−^ mice and 10 cochleae from five *Tmc2*^−/−^ mice (both genotypes: P4–P5 + 1 day *in vitro*). Below, we investigate whether different permeation properties of the MET channels for aminoglycoside antibiotics might also contribute to this marked difference in susceptibility. From about P6 onwards, a time when TMC1 expression rapidly increases in the cochlea (Kawashima et al., 2011), OHCs from *Tmc2*^−/−^ mice became gradually more susceptible to streptomycin, and similar to that observed in *Tmc2*^+/−^ cells (Figures 2D–F).

### TMC2 Reduces MET Current Block by Extracellular Dihydrostreptomycin

The aminoglycoside antibiotic DHS is a well characterized permeant blocker of the MET channel (Marcotti et al., 2005). The degree of block of the MET channel by DHS varies with extracellular Ca^2+^ and membrane potential (Kroese et al., 1989; Ricci, 2002; Marcotti et al., 2005), with high Ca^2+^ concentrations reducing both the entry of the aminoglycoside into the MET channel and the channel’s affinity for the drug, and positive membrane potentials reducing the degree of block of the current by DHS. These results led to the proposal that the DHS-binding site sits within the channel’s permeation pathway (Kroese et al., 1989; Marcotti et al., 2005; van Netten and Kros, 2007). Therefore, we assessed whether TMC2 directly affected the ability of DHS to block the MET channel. MET currents were recorded by stepping the membrane potential between −161 mV and +99 mV in 20 mV increments while displacing the hair bundles of *Tmc2*^+/−^ and *Tmc2*^−/−^ OHCs before and during the application of 30 μM extracellular DHS (only voltage steps to −81 mV and +99 mV are shown in Figures 3A,B). Extracellular DHS caused a voltage-dependent block of the MET current, with positive membrane potentials relieving the block in both genotypes (Figures 3A–D), as previously shown (Marcotti et al., 2005). The dose-dependence of the block of the MET current by DHS is shown in Figure 3E. At all concentrations tested, the degree of block of the MET current (*I*_DHS_/*I*_control_) at −81 mV in *Tmc2*^−/−^ OHCs was significantly stronger than in *Tmc2*^+/−^ OHCs (3 μM: *P* < 0.05; 10 μM: *P* < 0.005; 30 μM: *P* < 0.02). The concentration for half block (*K*_D_) at −81 mV in *Tmc2*^−/−^ OHCs (5.9 μM) was significantly lower (*P* < 0.001) than that measured in *Tmc2*^+/−^ OHCs (9.1 μM). We further investigated the voltage dependence of the block of the MET channel by DHS in OHCs by plotting the MET current in the presence of the aminoglycoside as a fraction of the control current (*I*_DHS_/*I*_control_, Figure 3F). The block of the OHC MET channel by DHS was consistently more effective in *Tmc2*^−/−^ mice compared to that in *Tmc2*^+/−^ mice and a partial relief of the block for large negative membrane potentials (negative to −80 mV) was seen in both genotypes (Figure 3F). This relief of the block is consistent with DHS being a permeant blocker of the MET channel when sufficient electrical driving force is present (Marcotti et al., 2005). The fits through the data are according to a two-barrier one-binding-site model (Marcotti et al., 2005).

To determine whether the rate of entry of DHS molecules into the OHCs changed in the presence of TMC2, we measured the time constant of MET current decline when exposed to DHS. To ensure that the MET channels are initially free from DHS, the hair bundles are first deflected in the inhibitory direction, closing all MET channels so that DHS is driven out of the permeation pathway, as the DHS molecules cannot reside in the closed channel (Marcotti et al., 2005). A saturating excitatory stimulus is then given prior to and during the application of different concentrations of extracellular DHS. In the presence of DHS the MET current is initially large, and then reduces to a steady level (Figures 4A,B) as the DHS enters and blocks the MET channel (open-channel block: Marcotti et al., 2005). Plotting the inverse of the time constant of DHS binding kinetics against the DHS concentration allows us to calculate the rate constant *k*_1_ (see “Materials and Methods” section) of DHS entry into the OHCs of both *Tmc2*^+/−^ and *Tmc2*^−/−^ mice (Figure 4C). Figure 4D shows that the absence of TMC2 increases the strength of *E*_b_, the DHS binding site in the channel pore. Because of the different numbers of MET channels between the two genotypes, and to take any possible difference in resting open probability out of the equation, we calculated entry rates of DHS per open channel, rather than per OHC. In the first instance we calculated entry for a 1 μM DHS concentration, as this is the approximate aminoglycoside concentration in the endolymph that just leads to ototoxicity *in vivo* (Tran Ba Huy et al., 1981), and for comparison with data from wild-type OHCs (Marcotti et al., 2005). Assuming a driving force of −150 mV and using 1.3 mM extracellular Ca^2+^, we estimated the rate of DHS molecules entering the OHC via the open MET channels to be 75.7 molecules/open channel/s for *Tmc2*^+/−^ and 63.9 molecules/channels/s for *Tmc2*^−/−^ OHCs. At −55 mV, near the resting potential of neonatal OHCs (Marcotti and Kros, 1999; Marcotti et al., 1999), the rates were 18.9 molecules/channel/s and 26.6 molecules/channel/s, respectively. These rates were comparable, but with some voltage dependence: in the absence of TMC2 they were somewhat lower at −150 mV and somewhat higher at −55 mV. At higher concentrations of DHS, when the entry was found to approach saturation, rates were consistently lower in the absence of TMC2. For 300 μM DHS, the same concentration that we used for the streptomycin experiments shown in Figure 2, entry rates reached 1078 molecules/channel/s for *Tmc2*^+/−^ and 586 molecules/channel/s for *Tmc2*^−/−^ OHCs at −150 mV. At −55 mV, rates were 315 molecules/channel/s for *Tmc2*^+/−^ and 74 molecules/channel/s for *Tmc2*^−/−^ OHCs. The lower saturation level for aminoglycoside entry in the absence of TMC2 is thus likely to contribute to the protection from ototoxic damage in the *Tmc2*^−/−^ mice during the first postnatal week, in addition to their smaller MET currents.

### TMC2 Reduces MET Current Block by Intracellular Dihydrostreptomycin

In order to determine whether the ability of DHS to block the MET current from the intracellular side was also affected in *Tmc2*^−/−^ OHCs, we added DHS in concentrations ranging from 30 μM to 3 mM into the intracellular solution. For these experiments the block of the MET current is only observed at positive potentials in OHCs from both genotypes (Figures 5A–C) but, as previously described (Marcotti et al., 2005), with a much reduced potency compared to extracellular DHS (Figure 3). Because of the difficulty of obtaining drug-free control recordings before and after intracellular drug application, we normalized the unaffected currents at −101 mV as a control to calculate the degree of block at +99 mV. We found that in P4–P5 OHCs the *K*_D_ for intracellular DHS block was significantly lower in *Tmc2*^−/−^ (124 μM) compared to that measured for *Tmc2*^+/−^ OHCs (717 μM, *P* < 0.00001) (Figure 5D). We also found that in *Tmc2*^+/−^ OHCs, the degree of block of the MET channel by 300 μM intracellular DHS (*I*_DHS_/*I*_c_), which corresponds to the steeper part of the dose-response curve, was significantly (*P* < 0.001) lower at P4–P5 (closed circles: Figure 5D) when compared to P8 (red triangle: Figure 5D). However, in *Tmc2*^−/−^ OHCs the degree of block was similar between P4–P5 (open circles: Figure 5D) and P8 (blue triangle: Figure 5D). The overlapping symbols at P8 also show that by this time the block was the same for *Tmc2*^+/−^ and *Tmc2*^−/−^ OHCs. This highlights that TMC2 confers a lower affinity of binding for DHS within the MET channel pore only during the first postnatal week when it is preferentially expressed over TMC1.

Vestibular hair cells, which, different from mouse cochlear hair cells, transduce low frequency signals, have been shown to retain TMC2 throughout adulthood (Kawashima et al., 2011). We hypothesized that TMC2 could be important for low frequency MET and as such it could remain in the low frequency region of the mature cochlea. Although the mouse cochlea is widely used for hearing research, it is mainly tuned to high frequencies (hearing frequency range: ~2–100 kHz, Ehret, 1975; Greenwood, 1990). To test our hypothesis, we performed MET current recordings from OHCs positioned in the apical coil of the gerbil cochlea, which is tuned to frequencies well below 1 kHz (mean *in vivo* characteristic frequency of ~0.35 kHz: Müller, 1996). Saturating bundle displacement of apical-coil gerbil OHCs (Figure 6A) elicited large MET currents similar to those obtained from mouse OHCs (Figure 1). In order to investigate whether TMC2 might be retained in older low-frequency gerbil OHCs, we tested the degree of block of the MET current by 300 μM intracellular DHS. At +99 mV a substantial block of the MET current was observed in apical OHCs of the gerbil (Figure 6B). The degree of block at +99 mV by intracellular DHS in gerbil P7–P8 OHCs (0.52 ± 0.07, *n* = 4) was not significantly different from that of mouse P8 OHCs of either *Tmc2*^+/−^ (0.57 ± 0.02, *n* = 7) or *Tmc2*^−/−^ (0.57 ± 0.04, *n* = 7) mice (Figure 6C). This suggests that, as for P8 mouse apical OHCs, TMC2 is not expressed in more mature gerbil OHCs, even in low-frequency regions of the cochlea.

### The Absence of TMC2 Does Not Affect the Maturation of OHC Basolateral Properties

**Figure 7:**
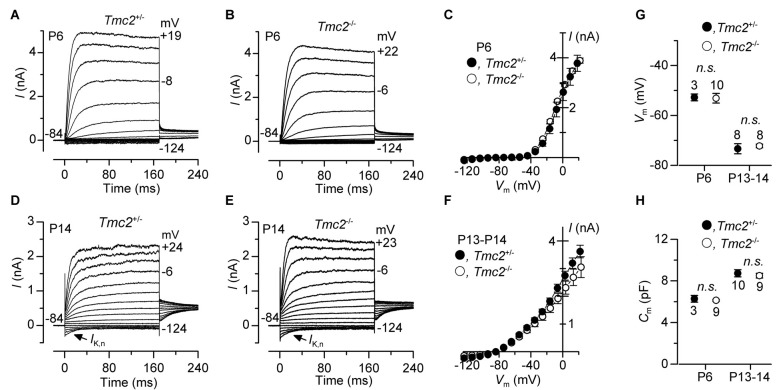
The development of basolateral membrane K^+^ currents in OHCs is not affected by the absence of TMC2.** (A–F)** Total K^+^ currents recorded from pre-hearing (P6: panels **(A–C)** and mature (P13–P14: panels **(D–F)** apical OHCs of *Tmc2*^+/−^ and *Tmc2*^−/−^ mice. Membrane currents were elicited in response to depolarizing voltage steps in 10 mV increments from –124 mV starting from a holding potential of –84 mV. Arrows in **(D,E)** indicate the presence of the negatively activating K^+^ current, *I*_K,n_, at P14. **(C,F)** show the current-voltage (*I*−*V*_m_) relation for the total outward currents at P6 (*Tmc2*^+/−^ *n* = 5, 3 mice; *Tmc2*^−/−^ *n* = 9, 2 mice) and P13–P14 (*Tmc2*^+/−^ *n* = 10, 5 mice; *Tmc2*^−/−^ *n* = 8, 5 mice). **(G,H)** Resting membrane potential (**G**, *V*_m_: P6* P* = 0.92; P13–P14 *P* = 0.62) and membrane capacitance (**H**, *C*_m_: P6 *P* = 0.59; P13–P14 *P* = 0.60) of *Tmc2*^+/−^ and *Tmc2*^−/−^ OHC at both P6 and P13–P14 (number of mice as in **C,F**).

The absence or mutation of *Tmc1* (*deafness* and *Beethoven* mice, respectively) has been shown to prevent the normal progression in hair cell maturation at the onset of hearing, such that they retain immature biophysical properties (Marcotti et al., 2006). Given the similarity between the *Beethoven* (*Bth*) mutation in *Tmc1* and the *Tmc2* knockout in terms of causing a reduction in MET channel Ca^2+^ permeability (see “Results” section: also see Pan et al., 2013; Corns et al., 2016), we investigated whether the absence of *Tmc2* affected the development of basolateral K^+^ currents in OHCs (Figure 7). Membrane currents in OHCs were elicited by applying depolarizing voltage steps in 10 mV increments from −124 mV starting from a holding potential of −84 mV. At P6, OHCs from both genotypes showed a delayed rectifier outward K^+^ current (*I*_K,neo_) (Figures 7A–C). *I*_K,neo_ exhibited a normal time course and voltage dependence as previously reported in normal mice (Marcotti and Kros, 1999), and its size, measured at 0 mV, was similar between *Tmc2*^+/−^ (2.69 ± 0.21 nA, *n* = 5) and *Tmc2*^−/−^ (2.74 ± 0.09 nA, *n* = 9, *P* = 0.82) OHCs (Figure 7C). At around P8, *I*_K,neo_ is gradually replaced by *I*_K,n_, which becomes the major K^+^ current expressed in mature mouse OHCs (Marcotti and Kros, 1999). *I*_K,n_ is an outward K^+^ current activated at hyperpolarized membrane potentials (Housley and Ashmore, 1992; Marcotti and Kros, 1999) and carried by KCNQ4 channels (Kubisch et al., 1999). We found that *I*_K,n_ was present in both *Tmc2*^−/−^ and *Tmc2*^+/−^ P13–P14 OHCs (Figures 7C,E). The size of *I*_K,n_, which was measured as the deactivating tail currents at −124 mV from the holding potential of −84 mV (difference between instantaneous and steady state inward currents: Marcotti and Kros, 1999), was similar between *Tmc2*^+/−^ (396 ± 45 pA, *n* = 10, P13–P14) and *Tmc2*^−/−^ (395 ± 53 pA, *n* = 9, P13–P14, *P* = 0.99). The total outward current measured at 0 mV (Figure 7F) was also similar between the two genotypes (*Tmc2*^+/−^: 2.6 ± 0.1 nA, *n* = 10; *Tmc2*^−/−^: 2.2 ± 0.2 nA, *n* = 8, P13–P14, *P* = 0.10). Moreover, all of the other biophysical properties of OHCs, including the resting membrane potential (*V*_m_, Figure 7G) and cell membrane capacitance (*C*_m_, Figure 7H) did not differ between *Tmc2*^+/−^ and *Tmc2*^−/−^ mice. This indicates that a loss of TMC2 appears to have no detrimental effect on the maturation of the basolateral membrane currents of OHCs, in contrast to what has previously been shown for the M412K point mutation in TMC1 (*Beethoven* mice: Marcotti et al., 2006).

## Discussion

The temporal expression of TMC2 (Kawashima et al., 2011) indicates a transient role for this protein during development of cochlear hair cells. Our study demonstrates that the permeant MET channel blocker DHS has an increased potency in *Tmc2*^−/−^ mice during the first postnatal week, indicating that TMC2 reduces the affinity of the MET channel for DHS. The absence of TMC2 in *Tmc2*^−/−^ mice, however, protects the OHCs from aminoglycoside ototoxicity. This protection was linked, in addition to the smaller MET current in OHCs from *Tmc2*^−/−^ mice, to the lower saturation level for DHS entry into the MET channel. In addition, we found that TMC2 confers a higher Ca^2+^ permeability, as previously described (Kim and Fettiplace, 2013; Pan et al., 2013; Beurg et al., 2015), in apical OHCs during the first postnatal week. Given its effects on the MET channel’s permeation properties, we propose that TMC2 is an intrinsic part of the MET complex in mammalian OHCs during the first postnatal week. Yet, the lack of functional deficits in both mature OHC physiology, observed in this study, and ABR measurements (Kawashima et al., 2011) from *Tmc2*^−/−^ mice, suggests that the expression of TMC2 may be an evolutionary redundancy within the mammalian cochlea. Alternatively, TMC2 could promote the initial growth of stereocilia (Vélez-Ortega et al., 2017) which, for the transducing stereocilia (shorter rows), occurs within the first few postnatal days (Tilney et al., 1992).

### TMC2 and TMC1 Are Components of the Neonatal MET Channel

Increasing evidence, including the localization of TMC1 and TMC2 to the site of the MET channel (Kawashima et al., 2011; Kurima et al., 2015), points to roles for both proteins as components of the MET channel. The M412K point mutation in *Tmc1* in IHCs (Pan et al., 2013) and OHCs (Beurg et al., 2015; Corns et al., 2016) reduces the MET channel’s permeation for Ca^2+^ ions. By contrast, TMC2 confers higher Ca^2+^ permeability (see also Kim and Fettiplace, 2013; Pan et al., 2013; Beurg et al., 2015) to the MET channel. Moreover, both proteins directly affect the strength of the binding site for the aminoglycoside DHS within the MET channel pore, with TMC2 (Figure 4D) and the M412K point mutation in *Tmc1* (Corns et al., 2016) both reducing the affinity of the DHS binding site in the channel pore. While the point mutation in *Tmc1* strongly affected the binding site in the permeation pathway for extracellular DHS, resulting in a reduced entry rate of the drug molecules into the hair cells, the effect of TMC2 was more subtle, with no clear consistent effect on drug entry for low concentrations of DHS. For higher, saturating DHS concentrations the presence of TMC2 increased drug entry during the first postnatal week compared to when the MET channels contained a greater proportion of TMC1 (from ~P5 onwards: Kawashima et al., 2011), pointing to different MET pore properties between the two phenotypes. The saturating DHS entry rate for the MET channel in OHCs from P5–P8 wild-type CD1 mice was 1527 molecules/open channel/s (calculated at −150 mV for 300 μM DHS and using 1.3 mM extracellular Ca^2+^: Marcotti et al., 2005). Despite the fact that at P5–P8 the normal MET channel contains little or no TMC2, the DHS entry, per open channel, was much larger than when TMC2 was not present in the first postnatal days (586 molecules/s: see “Results” section), but closer to when TMC2 was present (1078 molecules/s). Wild-type controls (P6–P8, apical coil) for the *Bth* mutant mice (*Tmc1*^+/+^: C3HeB/FeJ), gave a similar result to the CD1 mice, at 1462 molecules/s (calculated from data in Corns et al., 2016). The M412K point mutation in *Tmc1*^Bth/Bth^ mice (P6–P9) reduced the entry of 300 μM DHS to 521 molecules/s, similar to the TMC2-lacking channels in the first postnatal week. These findings suggest that there is a developmental change in the pore properties of MET channels containing TMC1 but not TMC2 subunits between the first and second postnatal weeks. This change, revealed by a low saturation level of DHS entry in the first postnatal week, is partly compensated for by TMC2.

Block by intracellular DHS, which appeared non-permeant over the voltage range that we could test, was more strongly reduced by the presence of TMC2 (Figure 5) than by the *Tmc1* point mutation, suggesting that structural differences between TMC2 and TMC1 are located at different positions along the proteins than the point mutation. The ability of TMC2 and TMC1 to directly alter the permeation properties, conductance, DHS binding site and resting open probability of the MET channel (for TMC1: Pan et al., 2013; Corns et al., 2016), not only supports a direct role for both in MET, but also the conclusion that these two proteins contribute to the properties of the permeation pore of the MET channel.

### The Physiological Relevance of TMC2

Different from the mammalian cochlea, vestibular hair cells retain TMC2 in their stereociliary bundle throughout adulthood, such that mechanoelectrical transduction in vestibular organs relies on both TMC1 and TMC2 (Kawashima et al., 2011). *Tmc2* transcripts are also observed in the adult inner ear of zebrafish (Maeda et al., 2014). From a functional point of view, one of the main differences between the auditory and the vestibular system is that vestibular end organs, such as the semicircular canals and utricle, respond to subacoustic stimuli up to a few tens or hundreds of Hertz, respectively, in both mammals and lower vertebrates (see: Wilson and Jones, 1979; Eatock and Lysakowski, 2006; Abbas and Whitfield, 2010). We found that the sensitivity to intracellular DHS of the MET current recorded from apical OHCs of the P8 gerbil cochlea (tuned in adult gerbil to ~350 Hz: Müller, 1996), the frequency range of which is near the upper limit of that of the vestibular end organs, was indistinguishable from that measured in aged-matched mouse OHCs (Figure 6). By this time TMC1 is already the main MET channel subunit in mice (Kawashima et al., 2011). This indicates that the expression of TMC2 is not a specific characteristic of hair cells detecting low frequency stimuli. Therefore, there must be another reason as to why TMC2 is no longer expressed in the functionally mature MET channel of the mammalian cochlea.

One of the characteristic features of the mature cochlea is the presence of a large endocochlear potential (~+90 mV) between the endolymph, which surrounds the mechanotransducer apparatus, and the perilymph present around the hair cell basolateral membrane (Békésy, 1952; Bosher and Warren, 1971). This potential is linked to the different ionic composition between the endolymph (K^+^: ~150 mM; Wangemann and Schacht, 1996; Ca^2+^: ~20 μM in the adult; Bosher and Warren, 1978) and the perilymph (Na^+^: ~150 mM, Ca^2+^: 1.3 mM; Wangemann and Schacht, 1996). However, in rodents, the endocochlear potential is absent or very small during the first postnatal week when the endolymph and perilymph are still similar in composition, and it only starts to increase to ~+15 mV during the second postnatal week (Bosher and Warren, 1971). This early low endocochlear potential is comparable to the endovestibular potential measured in mature vestibular end organs (a few mV: Schmidt, 1963). The physiological consequence of this small potential is that the electrochemical gradient that drives Ca^2+^ entry into hair cells is substantially reduced compared to that observed in the mature cochlea. The increased Ca^2+^ permeability conferred to the MET channel by TMC2 in vestibular and early postnatal cochlear hair cells is likely to compensate for this reduced driving force. This could be essential to enable sufficient Ca^2+^ to enter the hair cells to drive Ca^2+^-dependent processes such as adaptation of the MET channel (Corns et al., 2014; Marcotti et al., 2016), which is known to first appear in mice during a time when TMC2 is the main MET channel subunit (<P6). Calcium-induced adaptation in early postnatal OHCs is quite slow and comparable to that recorded in vestibular hair cells (Eatock, 2000; Géléoc and Holt, 2003). Conversely, in the adult mammalian cochlea, the combined endocochlear potential and the higher Ca^2+^ permeability conferred by TMC2 might produce excessive Ca^2+^ entry through the MET channel, reducing its resting open probability and the ability to function at its optimal operating range. This excessive Ca^2+^ influx into mature cochlear hair cells through the MET channels might then in turn lead to cytotoxicity and cell degeneration (Orrenius et al., 2003). An additional or alternative role for the increased Ca^2+^ permeability conferred by TMC2 might be to promote the initial growth of the transducing stereocilia (shorter rows) (Vélez-Ortega et al., 2017), which is known to occur within the first few postnatal days (Tilney et al., 1992).

We also found that different from TMC1 (Marcotti et al., 2006; Kawashima et al., 2011; Kim and Fettiplace, 2013), TMC2 was not required for the maturation of mechanoelectrical transduction and the basolateral properties of OHCs, which parallels previous evidence showing that type II cells of the utricle develop normally in its absence (Kawashima et al., 2011). This suggests that the presence of a functional MET channel during the first few postnatal days, when TMC2 expression predominates, has no bearing on hair cell maturation; whereas the ability of cochlear hair cells to mechanotransduce during the second postnatal week, when TMC1 expression predominates, is essential for their correct maturation into mature sensory receptors (Marcotti et al., 2006).

Recent studies have demonstrated that the auditory part of the inner ear evolved from the vestibular part (Gacek, 2009; Duncan and Fritzsch, 2012). Therefore, we propose that although TMC2 is present during the initial assembly of the MET channel in early postnatal cochlear hair cells, it may be an evolutionary remnant that is not required for the highly specialized hair cells of the mature mammalian cochlea.

## Author Contributions

All authors helped with the collection and analysis of the data. WM conceived and coordinated the study. LFC, CJK and WM wrote the article.

## Conflict of Interest Statement

The authors declare that the research was conducted in the absence of any commercial or financial relationships that could be construed as a potential conflict of interest.
